# Formation of CuO nano-flowered surfaces *via* submerged photo-synthesis of crystallites and their antimicrobial activity

**DOI:** 10.1038/s41598-017-01194-5

**Published:** 2017-04-21

**Authors:** Fumika Nishino, Melbert Jeem, Lihua Zhang, Kazumasa Okamoto, Satoshi Okabe, Seiichi Watanabe

**Affiliations:** 1 Graduate School of Engineering, Hokkaido University, N13, W8, Kita-ku, Sapporo, Hokkaido 060-8628 Japan; 2 Faculty of Engineering, Hokkaido University, N13, W8, Kita-ku, Sapporo, Hokkaido 060-8628 Japan

## Abstract

We report the fabrication of flower-like CuO nanostructured surfaces *via* submerged photo-synthesis of crystallites (SPSC), which requires only UV illumination in neutral water. In this paper, we discuss the reaction mechanism of the photochemical formation of the SPSC-fabricated CuO nanostructures in detail based on surface microstructural analyses and a radiation-chemical consideration with additional gamma-ray irradiation. Since the SPSC method for surface nanostructural fabrication can work at low temperatures at atmospheric pressure without using harmful substances, it is a potential fabrication method for green nanotechnology applications. In this vein, the antibacterial activity of the nano-flowered CuO surfaces was tested against Gram-positive (*Staphylococcus aureus*) bacteria and Gram-negative (*Escherichia coli* K12) bacteria, and the results demonstrate that the nano-flowered CuO nanostructures act as an effective antimicrobial agent.

## Introduction

Nanostructured metal oxides are known to have different optical, chemical and mechanical properties from the bulk materials. Among these, CuO as a p-type semiconductor (indirect bandgap of 1.2 eV^1^ and direct band gap of 3.2–3.3 eV^2–4^) is chemically stable, inexpensive, and abundant. Thus, it is attracting attention in new areas such as gas sensors^5–7^, catalysts^8^, lithium-ion battery electrodes^9^, and antimicrobial materials^10–12^. To date, the most common CuO nanofabrication methods are based on hydrothermal^2, 13^ and solvothermal methods^14^, which are known for manufacturing metal oxide nanostructure materials. Other previously reported methods are laser deposition^15^, chemical vapor deposition (CVD)^16^, electrochemical methods^17^, and microwave or ultrasonic irradiation methods^18^. However, these existing methods sometime involve deleterious factors such as contamination with impurities, use of strong acid or strong alkali solutions, high temperature and high pressure requirements, or vacuum environments. Therefore, an environmentally benign fabrication process at normal temperature and normal pressure is desired to obtain high-quality products via a low-cost method.

Submerged photo-synthesis of crystallites (SPSC)^19^ as a new green technology method has the potential to solve these problems. SPSC is a photo-synthetic method that can produce nanocrystals in neutral aqueous environments with UV or visible light. A previous study utilizing SPSC revealed the formation of ZnO flower-like nanorods by UV irradiation^19, 20^. This was achieved by the creation of a ZnO layer on the substrate by submersion in solution plasma and subsequent UV (λ = 365 nm) light irradiation in ultrapure water. The nanorod size increased with the extension of the UV irradiation time, and the shape changed from tapered ends to flat ends. In the pure water environment, the photochemical reaction is considered to contribute to the ZnO nanorod fabrication, as indicated following reaction: $Zn+{H}_{2}O+h\nu \to ZnO+{H}_{2}$. In addition, it was suggested that the formation of the ZnO nanoflowers by SPSC was accompanied by a photoradical reaction *via* water decomposition, and it was also confirmed that hydrogen is generated during the reaction^19^.

The present study on CuO crystal synthesis *via* SPSC is also conducted by irradiation with UV light in ultrapure water and elucidates the photochemical reaction mechanism involved in microstructuring the CuO nano-flowered surface *via* the SPSC method. Furthermore, to investigate the nanostructuring effect and as an application, we carried out antibacterial activity tests of nano-flowered CuO surfaces against Gram-positive (*Staphylococcus aureus*) bacteria and Gram-negative (*Escherichia coli* K12) bacteria.

## Results and Discussion

### SPSC for CuO nano-flowered surface fabrication

**Figure 1:**
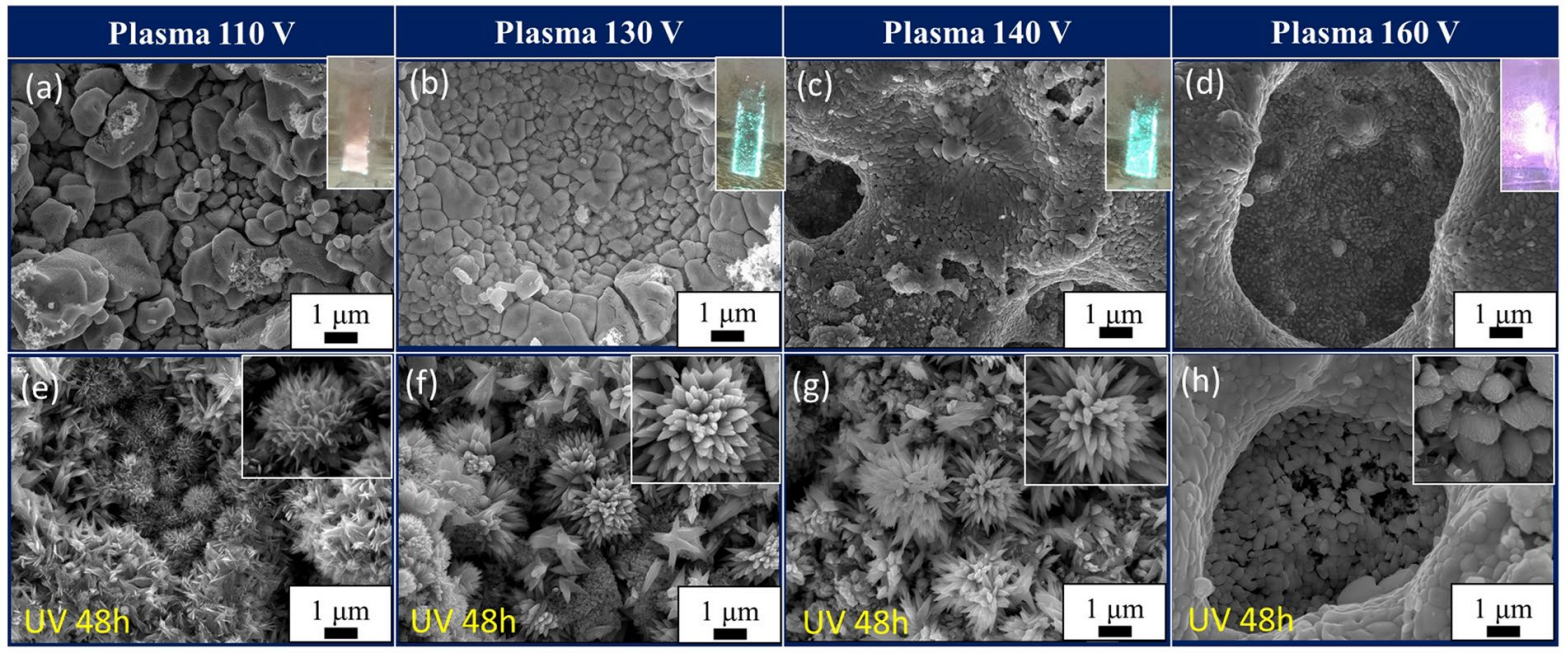
Surface morphology after SPSC of a Cu substrate plate. FE-SEM images of the sample surfaces (**a**–**d**) after solution plasma treatment at 110–160 V and (**e**–**h**) samples after 48 hours of UV irradiation followed by the plasma treatments above. Insets of (**a**–**d**) are photographs of samples during the plasma processing. Insets of (**e**–**h**) are enlarged views of each.

**Figure 2:**
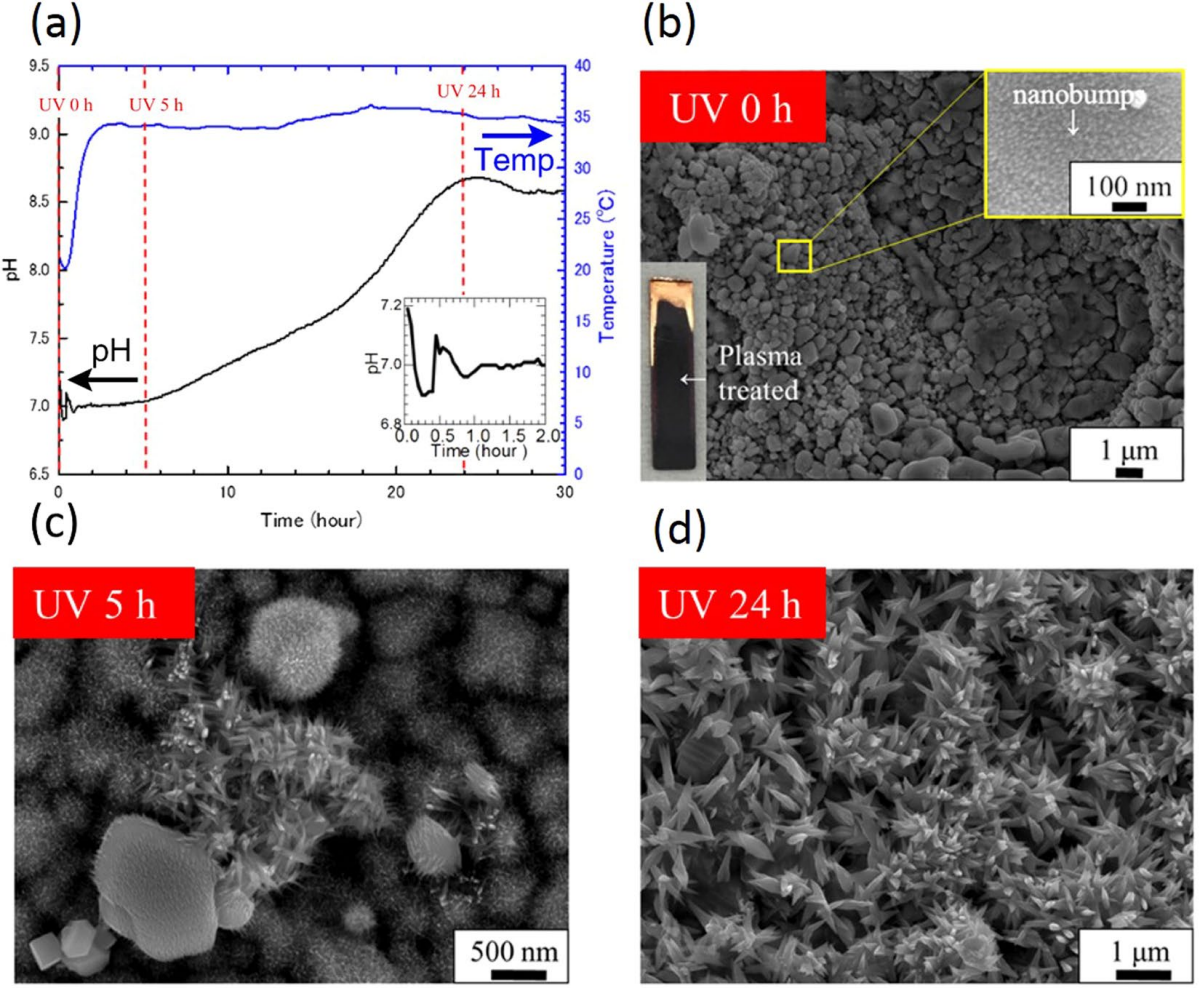
Photochemical reaction tracking on CuO formation. A pH-temperature graph during SPSC in (**a**) with respective UV irradiation times indicated by the red lines. Inset is the magnified time dependence pH during initial 2 hours UV irradiation. (**b**) FE-SEM image of the sample before UV irradiation. Inset is the enlarged image. Fine irregularities on the order of a few nanometers on the surface (nanobumps) can be observed. (**c**) Is fine fibrous crystals seen in samples after 5 hours of UV irradiation. (**d**) Is the sample after 24 hours UV irradiation; nanoflowers were formed on the surface.

Figure 1 shows the FE-SEM images of the surface subjected to UV irradiation after SPSC with the plasma treatment. In the solution plasma setup, plasma evolution is in accordance to high-low current and voltage increase (Supplementary Fig. S1). Glow discharge conditions with bluish-green light emission were achieved when the range of applied voltage was 110–140 V. Arc-discharge plasma with intense reddish-orange light emission formed when the applied voltage was >160 V. At 110 V, light emission could be observed from the bottom tip of the cathode. Here, the electric field became stronger with the cell voltage increase and led to the plasma spreading on the cathode surface. When subjected to 48 hours of UV irradiation, flower-like nanocrystals were formed on 110–140 V plasma-treated samples (Fig. 1(e–g)). There was no generation of such crystals on the surface of the >160 V arc-discharged samples (Fig. 1(h)). After glow discharge, metal-oxide seed irregularities (nanobumps) were formed (Fig. 2(b))^19, 21, 22^. Therefore, we choose the voltage range of 130–140 V as an optimized plasma-treatment condition for the following SPSC experiments.

In addition, from the point analyzed by EDS, components of the nanoflower were confirmed to have a CuO composition (Supplementary Fig. S2 and Table S1). As seen in the sample from the arc-discharge condition, when there was no formation of nanobumps on the surface of the sample after plasma treatment, nano-flower crystal formation after UV irradiation was not observed. The arc discharge yields a high conductivity because a large amount of electrons are supplied by thermionic emission from the heated cathode^21^. At that time, the current value becomes very high. If the material used as the cathode is a low melting point metal, the metal is melted instantly^21–23^. For this reason, nanobumps were not formed on the surface of the sample in the arc-discharge condition.

### Photochemical reaction mechanism of the CuO nanorod growth

Figure 2(a) shows a graph of the time dependence of pH and temperature during the SPSC. The black lines show pH values and the blue line shows the temperature. The water temperature increased steeply from 20 °C during the first 1–2 hours of UV irradiation. Then, it became constant at ~35 °C. At the same time, the initial pH value started to fluctuate from 7.0 during the first 1–2 hours of UV irradiation. The pH after 5 hours of irradiation was largely constant and then began to rise to become constant at pH = 8.5 after approximately 24 hours of irradiation.

FE-SEM images after 5 hours of UV and after 24 hours of UV are shown in Fig. 2(c,d). Fibrous crystallites of Cu(OH)_2_ were observed on the 5-hour UV-irradiated sample. Flower-like CuO crystals with tapered-end CuO nanorods were observed on the 24-hour UV-irradiated sample surfaces. However, in the case of without UV irradiation, i.e., only hydrothermal condition at elevated temperature, such nanoflower formation was not observed (Supplementary Fig. S3).

**Figure 3:**
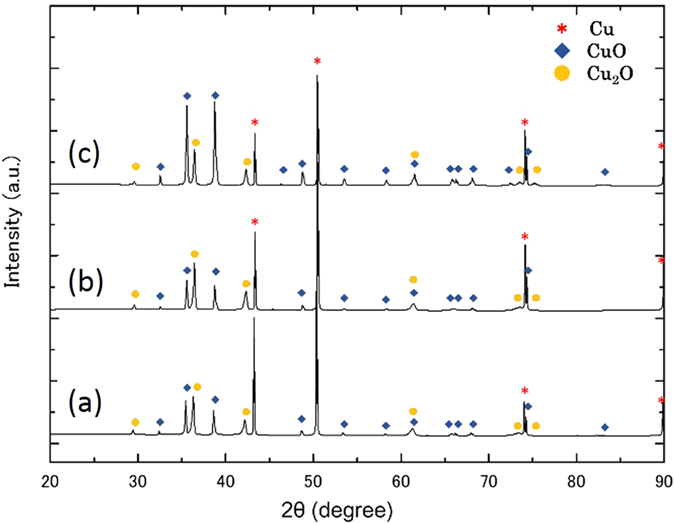
XRD patterns of the sample after plasma treatment and after UV irradiation. (**a**) Plasma treatment, (**b**) UV 5 hours, and (**c**) UV 24 hours.

**Figure 4:**
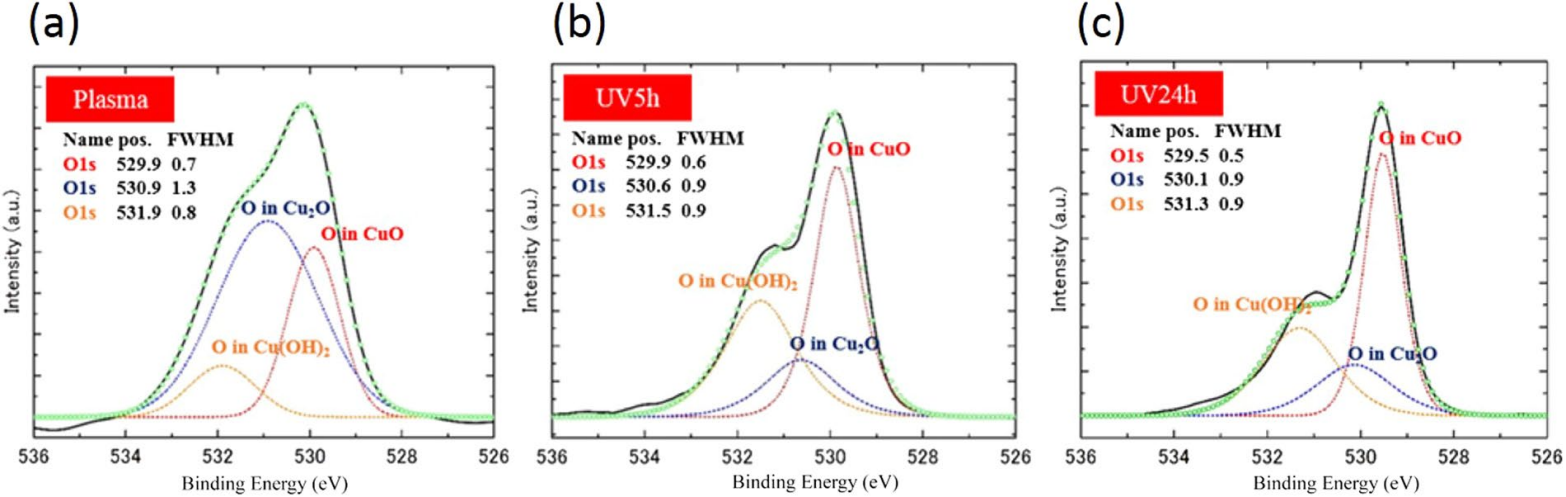
XPS spectra. XPS data of the sample after plasma treatment and after UV irradiation. (**a**) Plasma treatment, (**b**) UV 5 hours, (**c**) UV 24 hours.

The results of XRD at each stage are shown in Fig. 3. With the increase in UV irradiation time, the proportion of CuO increased. From these results, it was confirmed that the surface layer contained not only CuO but also Cu and Cu_2_O. No clear Cu(OH)_2_ peak could be observed in the XRD spectra. Thus, additional surface analysis by XPS was conducted. Figure 4 shows the results of the XPS analysis of the plasma-treated, UV 5 hour and UV 24 hour surface samples. Peaks at 529.5–530.2 eV correspond to CuO, while Cu_2_O peaks are at 530.2–530.8 eV. Cu(OH)_2_ is considered to have a peak at 530.9–531.5 eV. Each spectrum had confirmed peaks of Cu(OH)_2_, CuO, and Cu_2_O, and thus Cu(OH)_2_ was confirmed to be present on the surfaces. In addition, in the CuO and Cu(OH)_2_ peak intensity comparison of each sample, the CuO ratio increased with extended UV irradiation time, while the Cu(OH)_2_ showed a tendency to decrease. The ratio of Cu and O with increasing UV irradiation time was examined, approaching 1 after 24 hours of UV irradiation, suggesting that the reaction from Cu(OH)_2_ to CuO occurred during UV irradiation.

**Figure 5:**
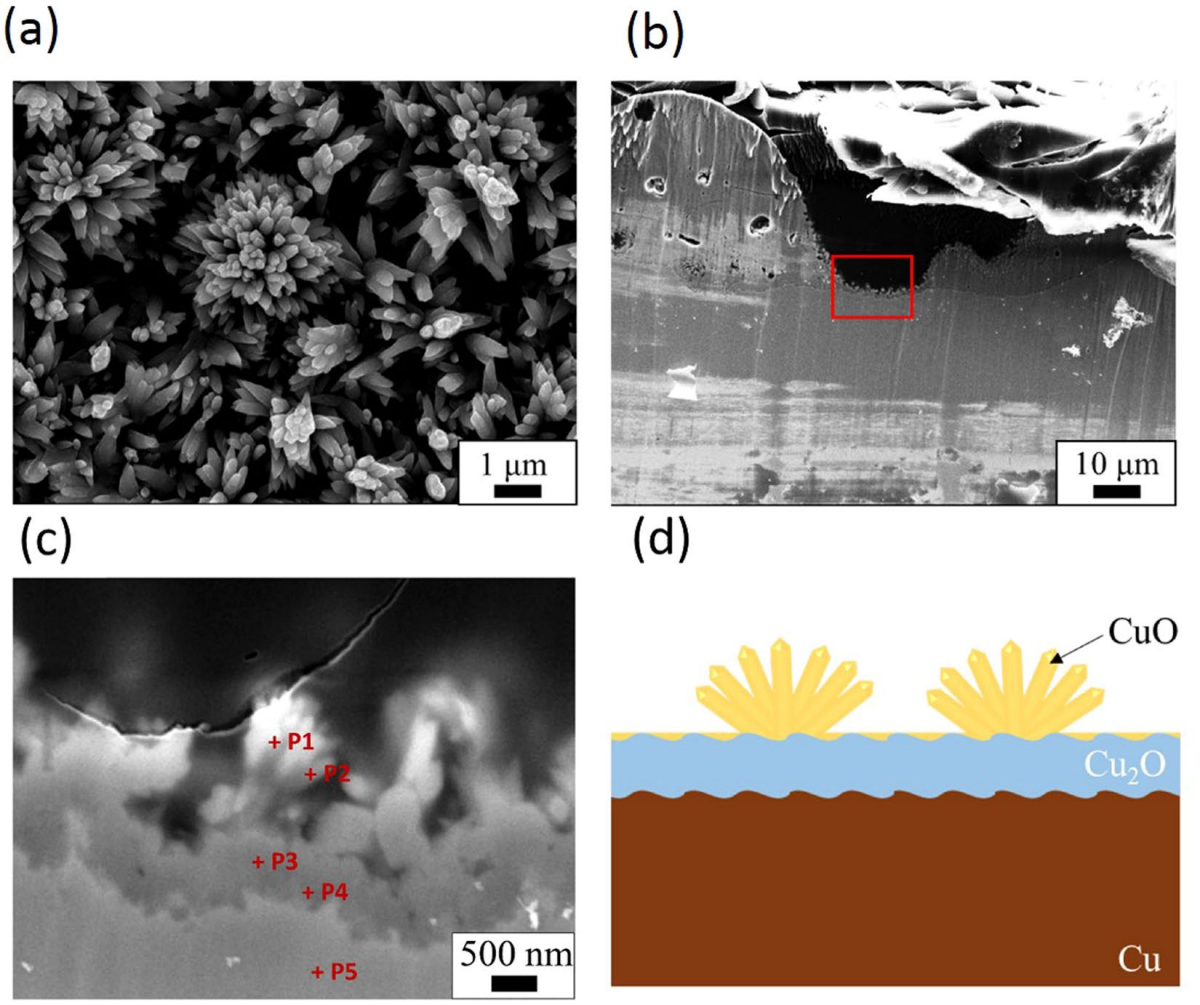
Cross-sectional images. (**a**) FE-SEM image of the sample surface after one week of SPSC. Nanoflowers were formed on the surface. (**b**) The cross-sectional FE-SEM image of (**a**) and (**c**) is the magnified view of the rectangular area in (**b**). P1-5 are the EDS point analysis position. The quantification result is indicated in Table 1. The black portion of the upper part is a resin that was used for cross-sectioning. (**d**) Schematic diagram of a cross-section of the samples prepared by the SPSC.

Figure 5 shows cross-sectional images of the samples after flower-like CuO nanocrystals formed on the surface. Figure 5(b,c) are the cross-sectional FE-SEM images. From these images, it can be seen that there are two layers; dark and bright inside the sample. The thickness of the dark part is approximately 0.8–1.0 μm, and a large number of growing rods were observed on it. These rods form nanoflowers. We compared the Cu and O components, eliminating the results of carbon in the quantitative analysis. From these results, the nanoflowers consist of CuO, while the darker part underneath is Cu_2_O formed during the plasma treatment; the lighter part is a Cu underlayer substrate. These results are consistent with the XRD results in Fig. 3. Since the black topmost layer of the sample’s surface consists of CuO after plasma-treatment and SPSC, hereafter, we consider only CuO formation rather than Cu_2_O.

**Table 1:** The EDS points analysis result for point P1-5 in Fig. 5(c).

	Cu (mol %)	O (mol %)
P1	51.1	48.9
P2	48.9	51.1
P3	61.9	38.1
P4	67.0	33.0
P5	100	0

**Figure 6:**
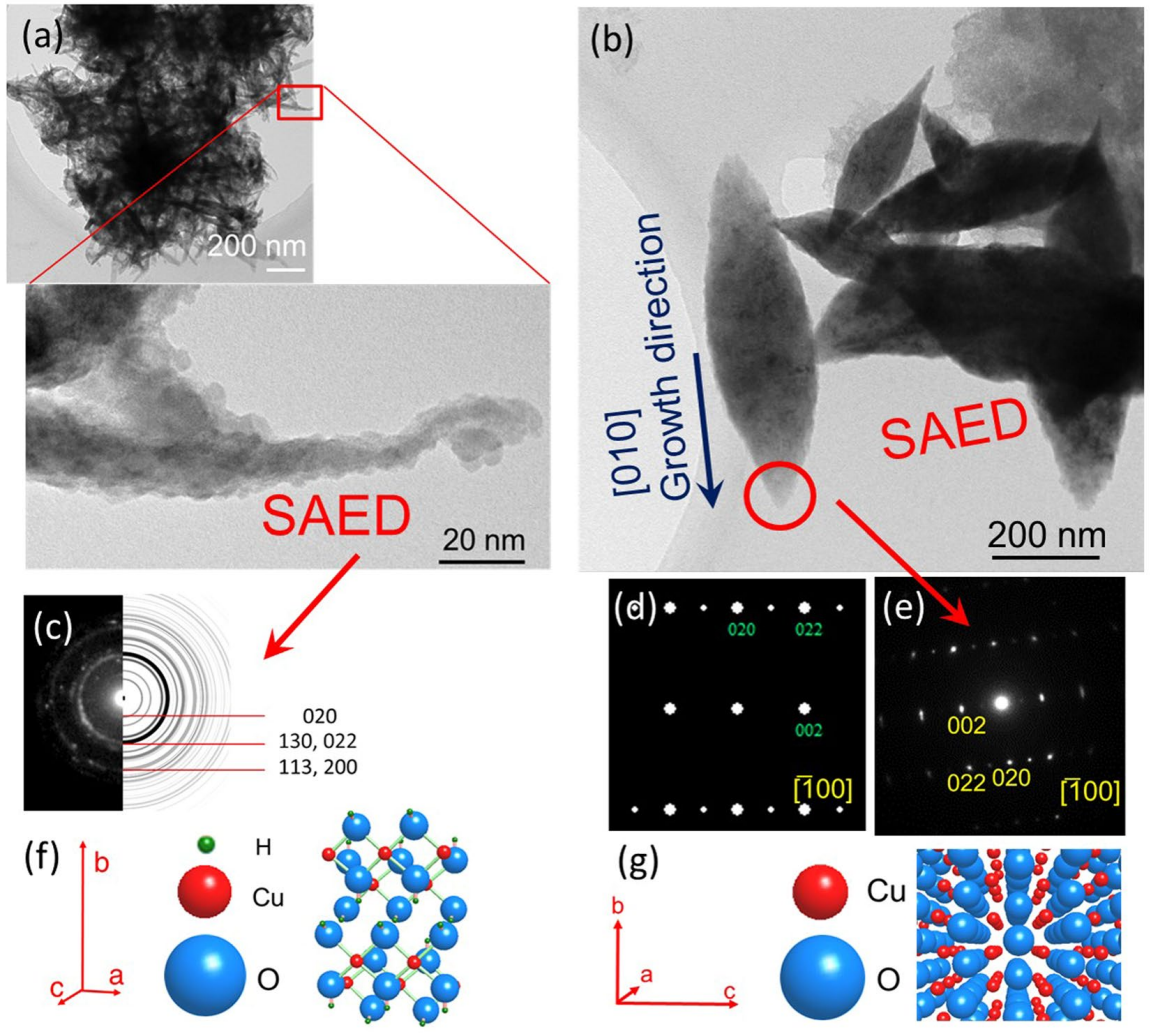
Structural characterisation of CuO crystals. TEM images of (**a**) bundled Cu(OH)_2_ particles and (**b**) leaves of CuO nanoflowers. The crystals of the oxide are considered to have peeled off in the growth process during UV irradiation. (**c**) Cu(OH)_2_ SAED ring pattern of the portion surrounded by a red square in (**a**) and calculated ring pattern of Cu (OH)_2_ on the right side. (**d**) Calculated electron diffraction pattern of CuO and (**e**) SAED pattern of the portion surrounded by a red circle of the CuO leaf in (**b**). (**f**) The crystal structure of Cu(OH)_2_: Orthorhombic pyramidal with lattice parameters of a = 0.295, b = 1.059, c = 0.5527 (nm). (**g**) The crystal structure of CuO: Monoclinic crystal with lattice parameters of *a* = 0.4684, *b* = 0.3423, *c* = 0.5129 (nm), α = 90°, β = 99.54°, γ = 90°.

TEM observations of the Cu(OH)_2_ and CuO nanocrystals are shown in Fig. 6. They were extracted from the ultrapure water after UV irradiation. There are large crystals of the two oxides that are fibrous and rice grain-like (Fig. 6(a,b)). A ring diffraction pattern observed in the SAED image of the fibrous crystal (Fig. 6(c)) indicated that the portion is made of fine crystals of Cu(OH)_2_. On the other hand, the SAED pattern obtained from leaf of the flower-shaped CuO nanorod (Fig. 6(e)) is consistent with the simulated CuO electron diffraction pattern. Therefore, in the growth process toward the CuO crystal leaf there are aggregated Cu(OH)_2_ particles, and it is thus inferred that the tapered CuO originated from an aggregation of Cu(OH)_2_ in water, followed by a light-driven dehydration reaction to single-crystal growth. Therefore, the CuO crystal is concluded to grow through a reaction of Cu → Cu(OH)_2_ → CuO, which is accompanied by a photochemical reaction.

**Figure 7:**
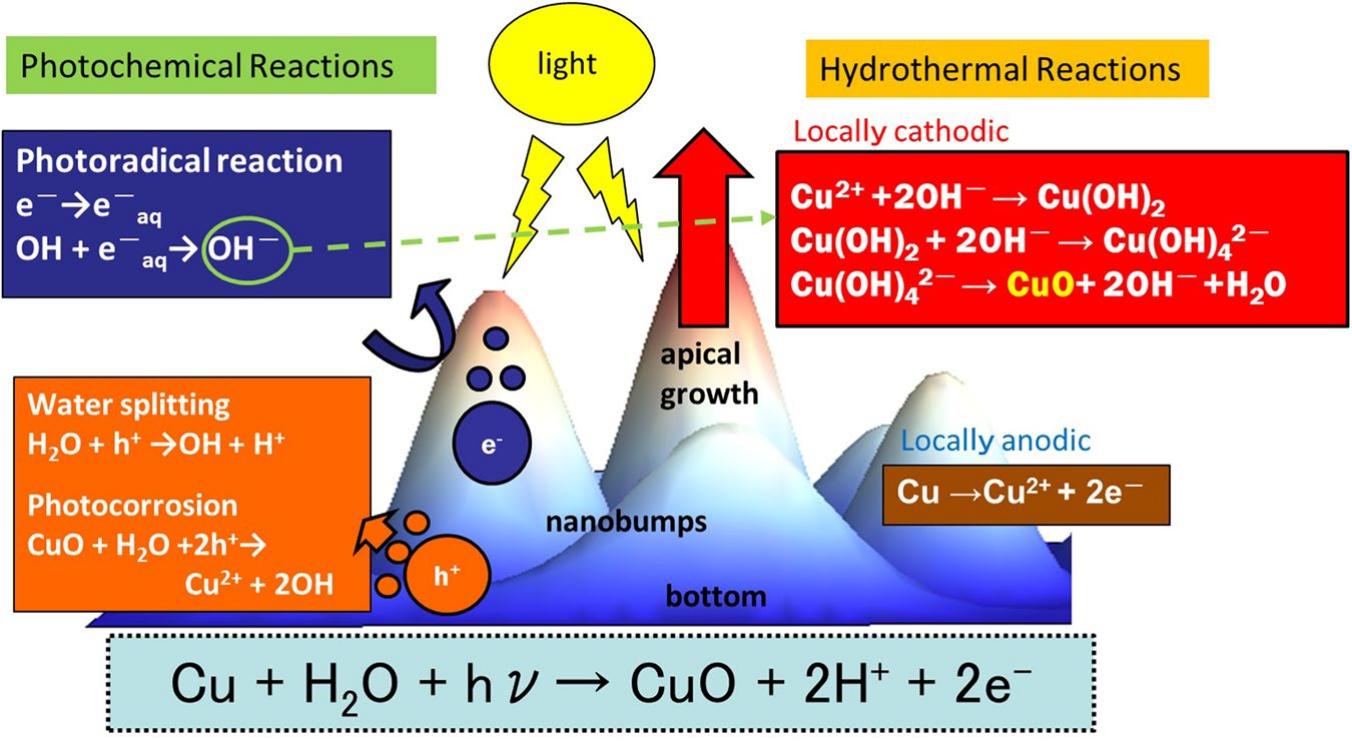
The mechanism of CuO crystal growth. A schematic view of the mechanism of CuO crystal growth in the SPSC process.

A schematic illustration of a growth reaction mechanism of CuO crystals by SPSC is shown in Fig. 7, in which both photochemical and hydrothermal reactions contribute to the SPSC^19, 20^. First, electrons and holes are generated by photosemiconductive reactions ($SC+h\nu \to SC({e}^{-}+{h}^{+})$) when UV light falls on nanobumps. Rather than recombination, some generated electrons and holes are separated. The holes move to the bottom of the concave nanobumps to create a local anode, while the electrons build up at the apical portion of the nanobumps to generate a cathodic environment^20, 24^. The photochemical reaction through water splitting (equation (1)) then builds holes at the bottom (anode), subsequently contribute to OH radical generation^25^ and/or to photocorrosion of CuO.

1 $${H}_{2}O+{h}^{+}\to OH+{H}^{+}\quad ({\rm{water}}\,{\rm{splitting}})$$

2 $$CuO+{H}_{2}O+2{h}^{+}\to C{u}^{2+}+2OH\quad ({\rm{photocorrosion}})$$

On the other hand, the electrons gathered at the tip, through the hydrated electron formation (equation (3)), induce the OH radicals to transform into OH^−^ ions and contribute to the generated alkaline atmosphere at the nanobump tip-end^19^.

3 $${e}^{-}\to {e}_{aq}^{-}\quad ({\rm{hydrated}}\,{\rm{electron}}\,{\rm{formation}})$$

4 $$OH+{e}_{aq}^{-}\to O{H}^{-}\quad ({{\rm{OH}}}^{-}\,{\rm{formation}})$$

These photoinduced reactions in water are believed to occur as photochemical reactions. The formation and reaction of transient radical species such as H, OH, ${{\rm{e}}}_{{\rm{aq}}}^{-}$, etc. occur within micro-second-order^26^. Nonetheless, local separation of OH^−^ at apical and H^+^ at the bottom occurs on the nanobumps surface (Fig. 7), which is assisted by the aforementioned morphology effect^19^. Otherwise, H_2_O will be immediately reproduced in the reverse reaction.

The resultant CuO nano-flower formation via SPSC is considered to be divided into the following three reaction stages^2, 27–29^. The first stage: immediately after putting the sample in water, it can be presumed that equation (2) or the following reaction of equation (5) occur at the bottom.

5 $$Cu\to C{u}^{2+}+2{e}^{-}$$

Equation (5) is a normal hydrothermal corrosion of the metal surface to yield Cu ions^28^, while equation (2) is the photocorrosion in water, if there is presence of oxide. In the present work, oxygen generation was not observed. This fact implies that the yield of oxygen will subsequently produce OH radical, as will be described later. Along with these reactions, pH values rise temporarily for approximately 1–2 hours (Fig. 2) then decreased. Through the OH^−^ generation (equation (4)), by water splitting described above, the following well known hydrothermal reaction to generate cupric hydroxide occurs^2, 29^.

6 $$C{u}^{2+}+2O{H}^{-}\to Cu{(OH)}_{2}$$

The second stage: After 5 hours of UV irradiation, the pH values rise again. The ${{\rm{OH}}}^{-}$ generation reaction in equation (4) causes the pH rise to alkaline levels, thereby generating the copper hydroxide complex ion as below^2, 27, 28^.

7 $$Cu{(OH)}_{2}+2O{H}^{-}\to Cu{(OH)}_{4}^{2-}$$

The third stage: The following CuO crystallization reaction then occurs^2, 27^.

8 $$Cu{(OH)}_{4}^{2-}\to CuO+2O{H}^{-}+{H}_{2}O$$

At this time, OH^−^ ions combine with Cu^2+^ in accordance with the above-mentioned reactions (6)–(8) to become CuO. Additionally, those light-driven reactions are induced dominantly at the tip-end, which is a factor of apical crystal growth^20^.

Accordingly, the net reaction representing CuO growth due to the SPSC is represented by the following.

9 $$Cu+{H}_{2}O+h\nu \to CuO+2{H}^{+}+2{e}^{-}$$

Here, unlike the case of zinc, hydrogen gas is not generated, which was confirmed by gas chromatography during the production of the copper oxide nanocrystals by SPSC. This is due to lower ionization tendency of Cu comparing with hydrogen and zinc (Cu^2+^ < H^+^ < Zn^2+^). Similarly to the polarization effect in voltaic cell where hydrogen deposition to a copper surface is inhibited, ${H}_{2}\to 2{H}^{+}+2{e}^{-}$ reaction occurs at the copper electrode surface owing to higher ionization tendency by hydrogen^30^. Based on our thermodynamic calculation results by HSC Chemistry software (Outokumpu Researh Oy, Pori, Finland), CuO formation (equation (9)) with hydrogen (H_2_) gas is unlikely to occur, because the required Gibbs energy (ΔG) is approximately 110 kJ, while in the case of ZnO formation, the ΔG value is negative (−81 kJ). This also suggested that the SPSC reaction stops if generated hydrogen cover the whole surface. Indeed, we did not observe crystal growth enhancement after more than 48 hours of UV irradiation, with less than a 0.1% weight increment (Supplementary Table S2). This value explains only the surface portion was modified. By refreshing the water, however, yielded the regrowth of CuO. We could fabricate micrometer-sized CuO flowers, as shown in Fig. 5(a), by sequential water refreshment, although crystallites that were peeled off in the water due to photocorrosion as in equation (2) were frequently seen.

### Comparison with gamma-ray irradiation and with SOD addition

**Figure 8:**
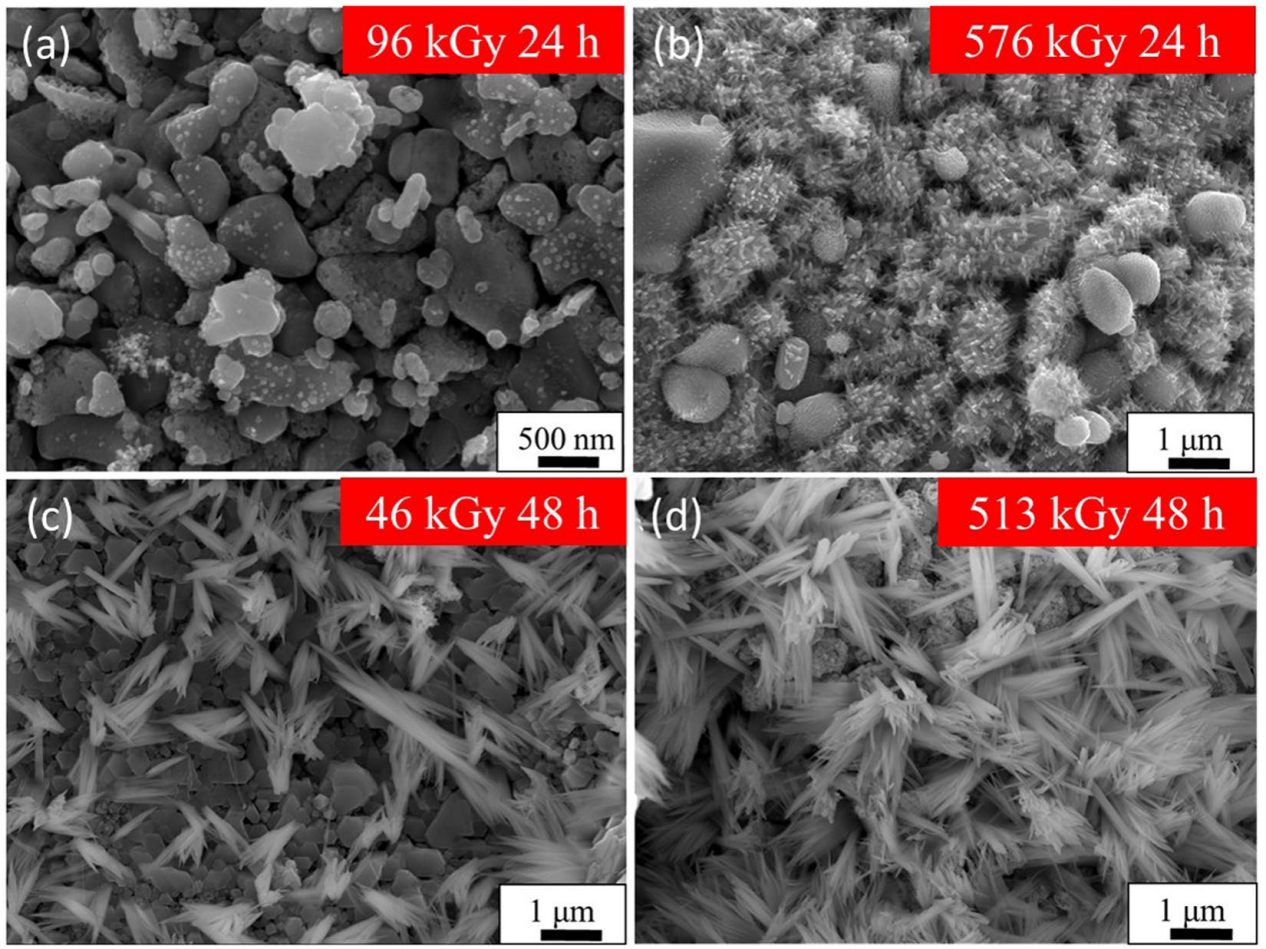
Gamma-ray irradiated substrates. FE-SEM images of the sample surface after gamma-rays irradiation at different fluences (96–576 kGy) and time (24 or 48 hours).

To confirm that SPSC involves a photoradical reaction, two additional experiments on the SPSC method were performed using gamma-ray irradiation and superoxide dismutase (SOD) addition. Instead of UV illumination, we carried out the gamma-ray irradiation experiment for the efficient formation of the radiolysis products of water such as OH, ${{\rm{e}}}_{{\rm{aq}}}^{-}$, H(H_2_), H_2_O_2_, and H_3_O^+^^26^. Figure 8 shows FE-SEM images of samples after irradiation with gamma-rays instead of UV. They were irradiated under various conditions by varying the irradiation time and the dose rate. Although the sample that received 24 hours of gamma-ray irradiation with a dose of 96 kGy (hereafter, such samples are denoted as 96 kGy 24 h) showed no crystal formation, it was confirmed that the 46 kGy 48 h samples generated fine acicular CuO crystals. Comparing the 576 kGy 24 h and 513 kGy 48 h samples, the higher dose rate (but shorter time) 576 kGy samples showed less crystal growth. Above a certain dose that the nanocrystal generation requires, the irradiation time has been found to influence the growth of the crystal size. By a radical reaction due to the gamma-ray radiation of high linear-energy-transfer (LET) radiation^26^, it is also possible to induce crystal submerged synthesis by higher energy photon irradiation.

It is important to note that dissolved oxygen gas can also enhance CuO formation during SPSC. It is well known that dissolved oxygen increases the concentration of H_2_O_2_, H_2_ and superoxide anion (${{\rm{O}}}_{2}^{-}$) in gamma-ray irradiated aerated water^31^.

10 $${O}_{2}+{e}_{aq}^{-}\to {O}_{2}^{-}$$

${O}_{2}^{-}$ is formed between O_2_ and ${{\rm{e}}}_{{\rm{aq}}}^{-}$.

11 $$OH+{O}_{2}^{-}\to {O}_{2}+O{H}^{-}$$

12 $${H}_{2}{O}_{2}+{e}_{aq}^{-}\to OH+O{H}^{-}$$

Hence, ${{\rm{O}}}_{2}^{-}$ reduces the OH radical to OH^−^ (equation (11)). Additionally, dissociative electron attachment of H_2_O_2_ on the adjacent surface contributes to OH radical and OH^−^ generation (equation (12)). These reactions may enhance the reactions in equations (6) and (7) because the local OH^−^ concentration increases. Therefore, CuO growth during SPSC is enhanced in aerated water.

**Figure 9:**
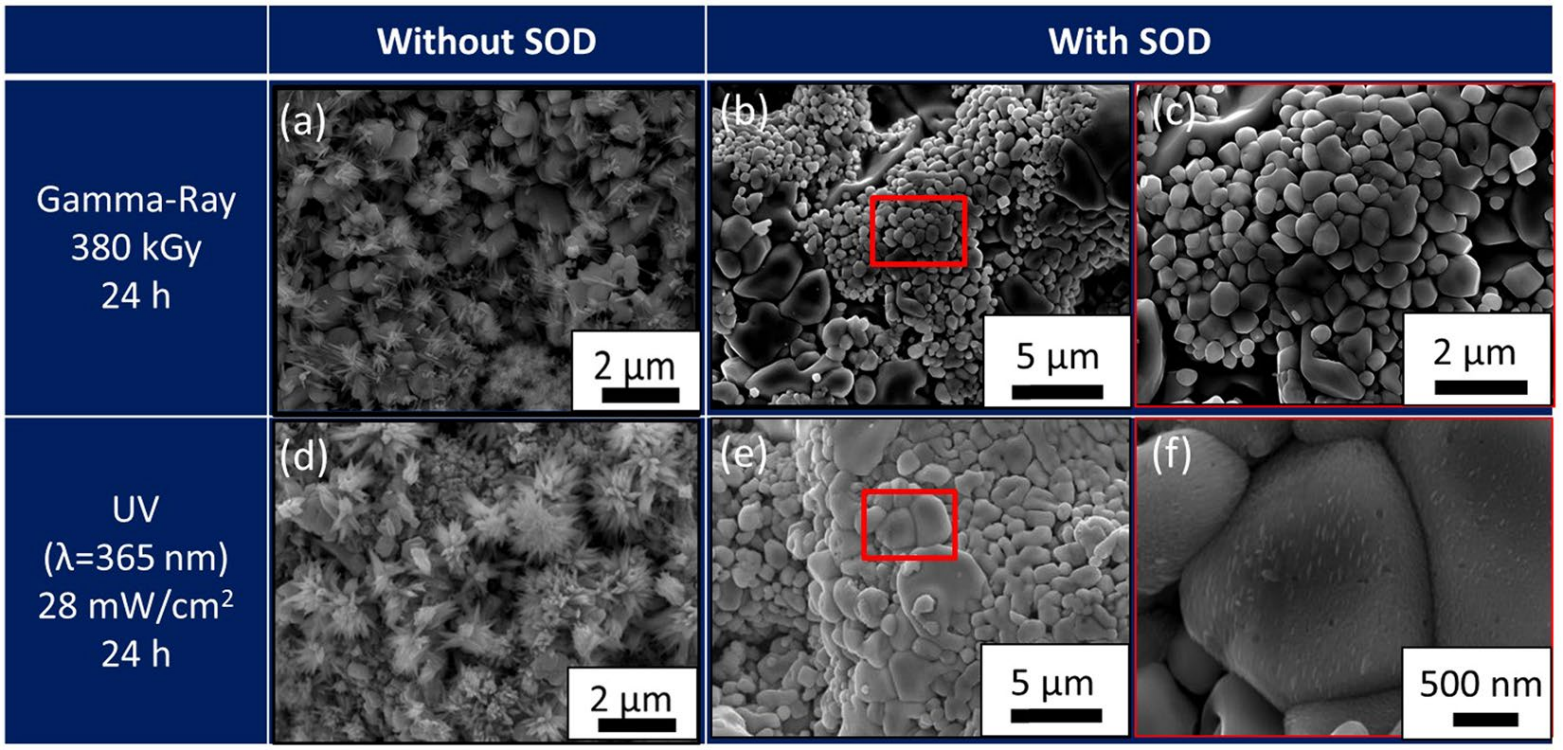
SOD addition test. FE-SEM images of the surface after irradiation of (**a**) 24 hours at 380 kGy gamma-rays without SOD in ultrapure water. (**b**) 380 kGy gamma-rays irradiation with SOD in ultrapure water. (**c**) Enlarged view of rectangle area in (**b**). (**d**) UV irradiation for 24 hours. (**e**) UV irradiation for 24 hours with the addition of SOD. (**f**) Enlarged view of rectangle in (**e**).

Figure 9 shows an FE-SEM image of UV- and gamma-ray-irradiated samples mixed with the SOD reagent in ultrapure water to scavenge ${{\rm{O}}}_{2}^{-}$. The SOD reagent is known to capture ${{\rm{O}}}_{2}^{-}$ ^32, 33^. Comparing the samples with and without SOD reagent addition, the samples without the addition of SOD show generated crystals, while in the samples with added SOD, the crystal formation is suppressed. This result suggested that ${{\rm{O}}}_{2}^{-}$ plays an important role in the CuO growth. From the results of these two additional experiments, the crystalline production *via* SPSC was revealed to be caused by photolysis or radiolysis products in oxygenated water.

### Antimicrobial activity test

**Figure 10:**
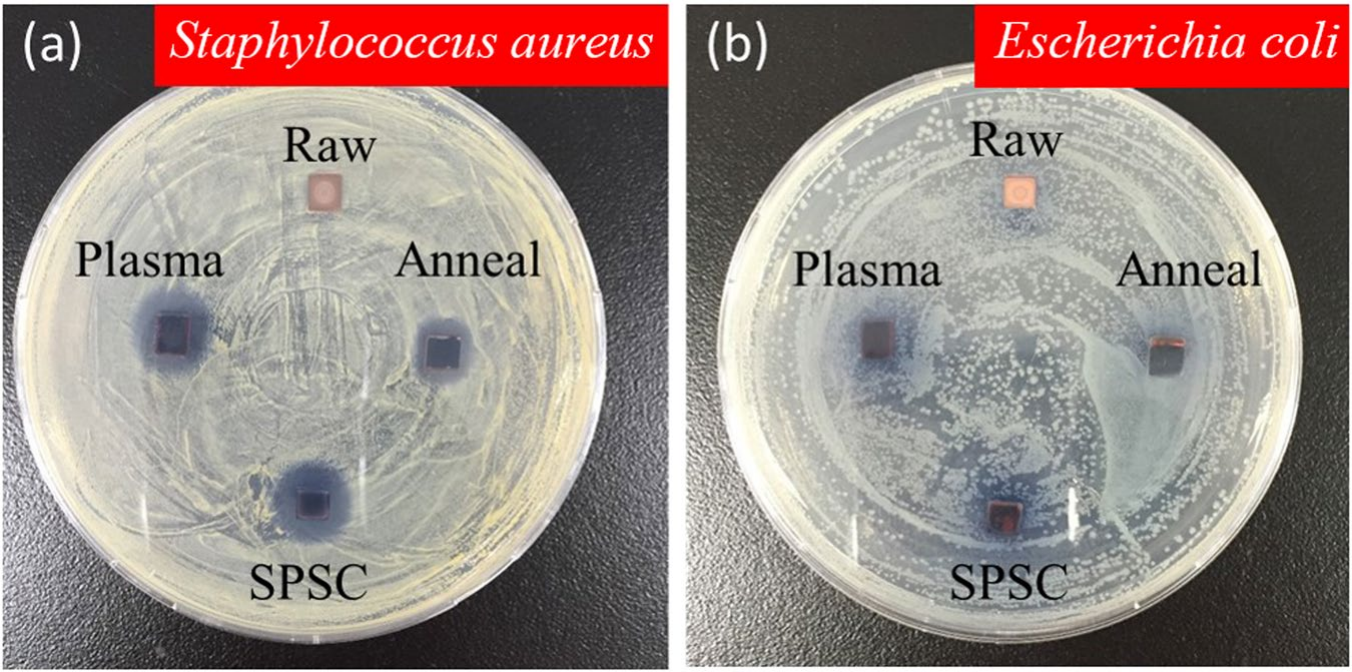
Antimicrobial activity test. Photos after culture with (**a**) Gram-positive (*Staphylococcus* *aureus*) bacteria and (**b**) Gram-negative (*Escherichia coli* K12) bacteria in agar in a Petri dish for 24 hours at 37 °C. The samples are raw (as-received Cu plate), plasma-treated, SPSC-UV48 h and annealed CuO.

As an illustrative application, we carried out an antibacterial activity test of nano-flowered CuO surfaces against Gram-positive (*Staphylococcus aureus*) and Gram-negative (*Escherichia coli* K12) bacteria. After 24 hours of incubation at 37 °C, clear inhibition zones were observed around the foil samples of CuO-annealed (see Supplementary Fig. S4), CuO-plasma and flowered CuO formed by SPSC (Fig. 10). The average inhibition zones for *Staphylococcus* were 1.0 cm for CuO-annealed, 1.2 cm for CuO-plasma and SPSC-CuO, and 0 cm for raw-Cu. The *Escherichia coli K12* average inhibition zones were 1.0 cm for CuO-annealed, 1.1 cm for CuO-plasma and SPSC-CuO, and 0 cm for raw-Cu. From these results, we observed that the antimicrobial activities of plasma-treated CuO and flower-shaped SPSC-CuO were higher than CuO-annealed. Furthermore, the plasma-treated CuO and SPSC-CuO had higher antibacterial activity against *S. aureus* than *E. coli*. This different sensitivity could be attributed to differences in the bacterial cell structures. *Staphylococcus* is composed of a thick peptidoglycan layer susceptible to intracellular transduction causing cell wall disruption.

In contrast, *E. coli* has a thin peptidoglycan layer and phospholipid bilayer cell membranes, which could be less prone to being damaged by CuO^34^. However, free Cu^2+^ ions are able to produce reactive oxygen species (ROS) such as ${{\rm{O}}}_{2}^{-}$, OH radical and H_2_O_2_, which cause disruptions to amino acid syntheses, resulting in bacterial death^28, 35^. Through our experiment, as the test plates (Fig. 10) were placed under fluorescent light, excited electron-hole pairs in CuO to generate Cu^2+^ is also possible in photocatalytic process. Focusing on CuO and Cu_2_O, the 700 °C annealed sample (CuO-annealed) conceivably exhibited Cu^2+^ effect from CuO on the basis of its XRD spectra (Supplementary Fig. S4) and near value of inhibition zones with SPSC-CuO and CuO-plasma. In addition, black surface of the CuO-annealed implies that CuO is more dominant than Cu_2_O on the surface. Cu(OH)_2_ is assumed to be least inactive species to contribute in antibacterial performance.

Relative to the CuO samples, such reactions rate increase with SPSC-CuO samples surface area due to nanosize effects. The Brunauer-Emmett-Teller (BET) analysis results indicated specific surface area for Raw-Cu, plasma-treated CuO, and SPSC-CuO were 6.8 × 10^−2^ (m^2^/g), 4.1 × 10^−1^ (m^2^/g), and 1.8 (m^2^/g), respectively. It is noteworthy that the samples used for BET measurement were plate shape bulk (20 × 5 × 0.5 mm). As previously mentioned, only the surface portion was modified via SPSC by referring to <0.1% of total sample weight increase. Thus, the present BET results can only conceivably yield much smaller than the logical value from oxide nanoparticles portion alone. Nonetheless, the ratio of specific surface area is 1.0: 6.0: 26.4, which indicates the specific surface areas of the CuO plate increased greatly after plasma treatment and SPSC process.

In some cases, Cu metal itself has a better antibacterial activity than CuO because of better electron acceptation in Cu^36^. As an example, a direct electron transfer between bacteria (negative) and Cu (electron acceptor) can best describe antibacterial activity through disruption of the cell membrane. However, our relatively smooth surface of raw-Cu sample exhibited least inactive antibacterial activity (0 cm inhibition zone). Therefore, we deduced that Cu^2+^ from CuO was responsible for the antibacterial performance.

Hence, the differences in the antimicrobial activities of different CuO samples might reflect their ROS production capability in the agar plates. In summary, nanostructured surfaces of SPSC-CuO exhibit high antibacterial activities.

## Methods

### Materials and surface pretreatment

The substrate material was a Cu plate (99.99%+) with dimensions of 35 × 5 × 0.5 mm (Nilaco, Tokyo, Japan). We carried out the surface pretreatment by the solution plasma technique^21, 37^ (Supplementary Fig. S1) under atmospheric pressure. The setup consisted of two electrodes immersed in a 300 mL glass beaker filled with a 0.1 M potassium carbonate (K_2_CO_3_) (pH = 11.5) aqueous solution. The anode was φ1 mm platinum wire (Nilaco, Japan) framed to a glass mesh. The target Cu plate was wrapped with φ0.5 mm Cu wire (Nilaco, Japan) to prepare it as a cathode. Glow discharge plasma was initiated on the Cu plate surface in the voltage range of 110–160 V using a direct current power supply (KIKUSI, PWR1600H, Japan) and maintained for 10 minutes. After the plasma treatment, the substrate was washed with deionized water and dried in ambient air. Before UV irradiation, the sample was cut to a 25 mm length.

### UV irradiation

The plasma-treated Cu plate was immersed into a polymethylmethacrylate (PMMA) cuvette filled with 4 mL of ultrapure water (Wako Pure Chemical, Japan)^19^. The pH and resistance of the ultrapure water were 7–7.5 and 18.2 MΩ·cm, respectively. Then, SPSC was conducted using a 100 W UV lamp (λ = 365 nm, 3.4 eV) (UVP, B-100AP, USA) for illumination inside a lightproof chamber. The distance between the cuvette and the UV lamp was 10 cm, yielding a typical intensity of 28 mW/cm^2^. Changes in the ultrapure water pH were measured using a pH meter. Experiments and data recording were performed at room temperature.

### Gamma**-**ray irradiation

The metal piece was immersed and sealed in 3 mL of ultrapure water (18.2 MΩ·cm) and was irradiated with gamma rays from ^60^Co at Osaka University ISIR. The absorbed dose was calculated by Fricke dosimetry.

### SOD test

Superoxide dismutase (SOD) is known to capture superoxide anions to suppress radical reactions^32, 33^. Experiments were performed to confirm the presence of a radical species upon UV irradiation or gamma irradiation by a 3 μL addition of SOD1 (1 mM).

### Surface observation and analysis

Field emission scanning electron microscopy (FE-SEM, JSM-7001FA, JEOL) was used to observe the specimen surface. X-ray diffraction (XRD, Rigaku, Tokyo, Japan, RINT2500HLB) with a Cu Kα line of 1.5406 Å was performed. A scanning field of 5° ≤ 2θ ≤ 100° was used, and peak fitting was performed in reference to CuO (JCPDS card no. 89-5897), Cu_2_O (no. 71-3645) and Cu (no. 89-2838). Transmission electron microscopy (TEM) and selected area electron diffraction (SAED) patterns for the crystal were obtained using a conventional-TEM (JEM-2000FX, JEOL) operated at 200 kV. The diffraction patterns were calculated using Mac TempasX (Total Resolution LLC). X-ray photoelectron spectroscopy (XPS, JEOL, JPS-9200) was used for the surface elemental analysis. In addition, a cross section polisher (CP, JEOL, IB 09010CP) was used for cross-sectional observation.

### Antimicrobial activity test

The antibacterial activity of CuO was demonstrated by the inhibition zone method using Gram-positive (*Staphylococcus aureus*) and Gram-negative (*Escherichia coli* K12) bacteria^10–12^. *S. aureus* and *E. coli* K12 were pre-cultured in nutrient broth (1 g peptone, 0.3 g NaCl, and 1 g beef extract in 100 mL of distilled water) overnight in a rotary shaker at 37 °C. The nutrient agar plates were prepared by adding 2 g of agar to the nutrient broth, autoclaving at 120 °C, and pouring into Petri dishes. Each overnight culture (the cell concentration was adjusted to 10^7^ cells/mL) was spread on the surface of the solidified agar plates using a sterile glass hockey stick. The test pieces were placed lightly on top of the agar medium. For comparison with SPSC-CuO, an annealed copper sample having a uniform CuO surface was also prepared by annealing for 3 hours at 700 °C in an open furnace. The plates were then incubated at 37 °C for 24 hours under fluorescent light, and bacterial growth inhibition levels were examined.

## Electronic supplementary material

Supplementary Information
