# Supplementary material for: Formation of CuO nano-flowered surfaces via submerged photo-synthesis of crystallites and their antimicrobial activity
Source: Sci Rep. 2017 Apr 21;7:1063. doi: 10.1038/s41598-017-01194-5 (PMC5430675; doi:10.1038/s41598-017-01194-5)
Supplement: Supplementary file 1 — Supplementary Information [file 41598_2017_1194_MOESM1_ESM.pdf]

Supplementary Information

Formation of CuO nano-flowered surfaces *via* submerged photo-synthesis of crystallites and their antimicrobial activity

Fumika Nishino[1], Melbert Jeem[1], Lihua Zhang[2], Kazumasa Okamoto[2], Satoshi Okabe[2], Seiichi Watanabe[2*]

1. Graduate School of Engineering, Hokkaido University, N13, W8, Kita-ku, Sapporo, Hokkaido, 060-8628, Japan.

2. Faculty of Engineering, Hokkaido University, N13, W8, Kita-ku, Sapporo, Hokkaido, 060-8628, Japan.

*sw004@eng.hokudai.ac.jp

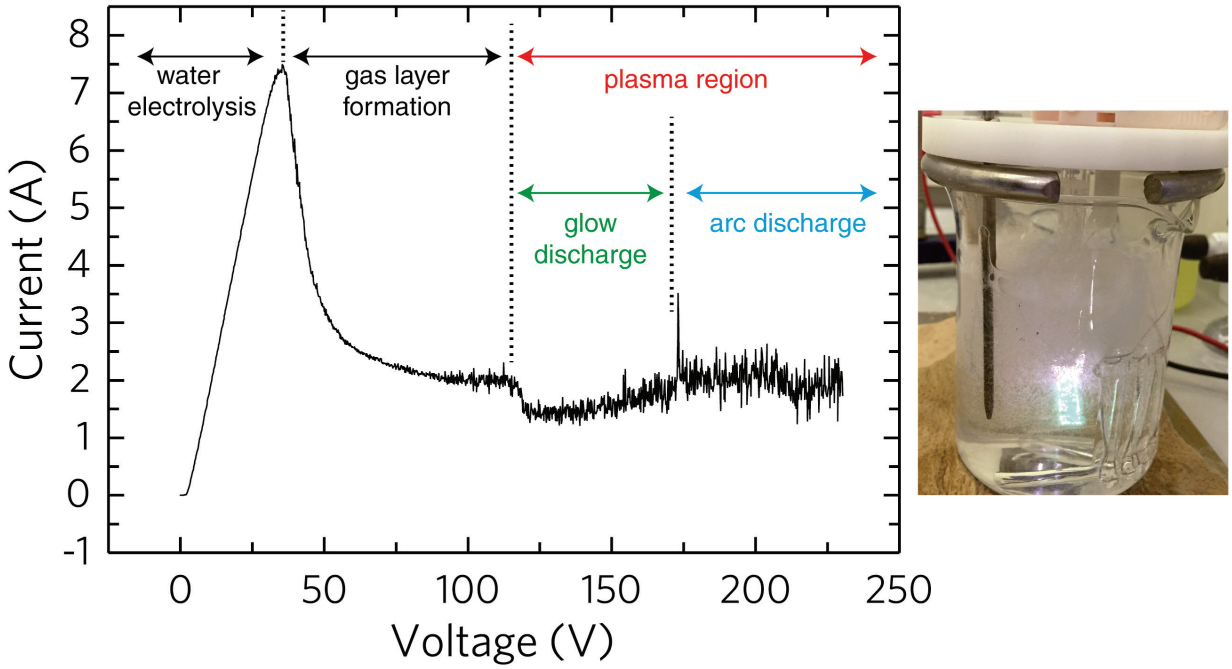

Supplementary Figure S1. Typical current-voltage relation curve during plasma treatment of Cu plate. On the far right is a photograph taken during glow discharge state of Cu.

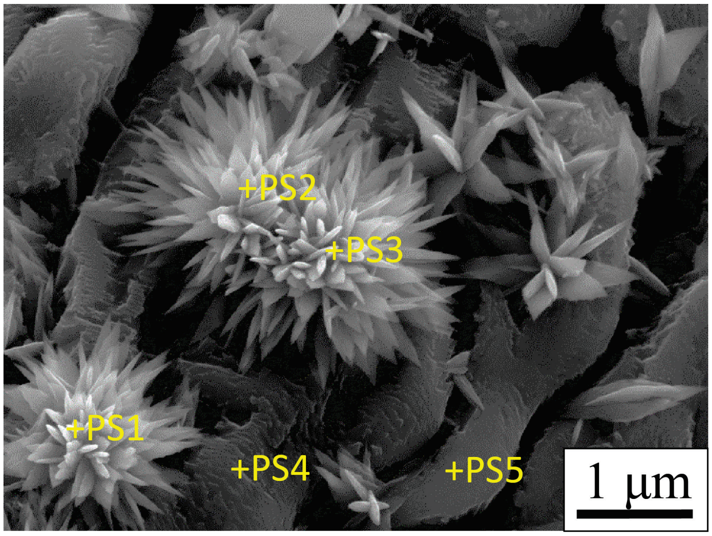

Supplementary Figure S2. The plan-view FE-SEM image of SPSC-24h sample and PS1-PS5 are the EDS point analysis position.

	Cu (mol %)	O (mol %)
PS 1	50.2	49.8
PS 2	45.7	54.3
PS 3	49.3	50.7
PS 4	74.3	25.7
PS 5	71.3	28.7

Supplementary Table S1. The EDS points analysis result for point PS1-PS5 in Figure S2.

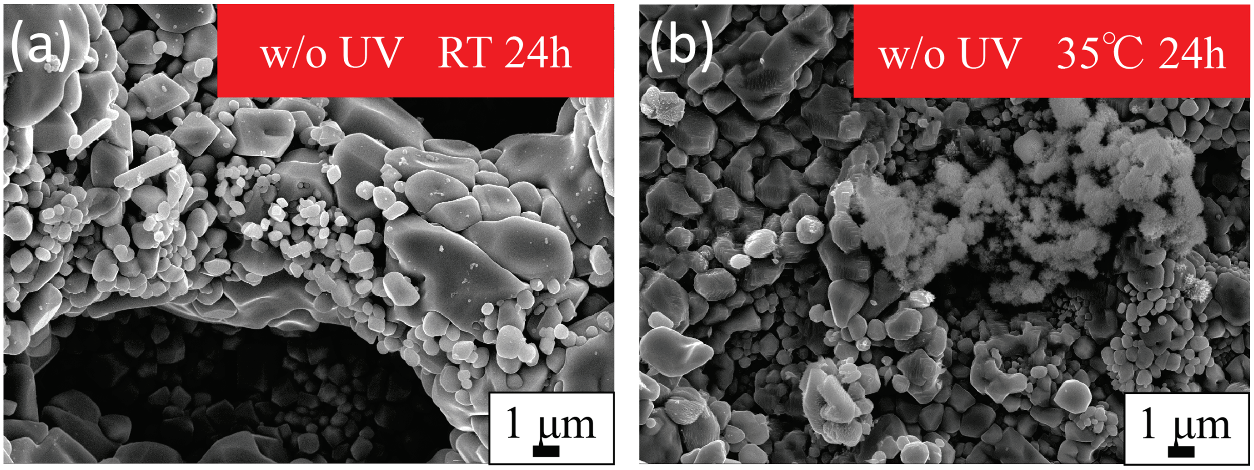

Supplementary Figure S3. Thermal effect on CuO nanostructure formation without UV irradiation for 24 hours in water. FE-SEM images (**a**) at room temperature (**b**) at 35 °C. No nanoflower formation was observed in both cases.

Sample	Before UV (mg)	After UV (mg)	Weight change (mg)	Weight change (%)
Plasma-treated	985.487	985.848	0.361	0.037
Without plasma (raw)	1558.610	1559.011	0.401	0.026

Supplementary Table S2. Typical weight change of Cu samples after UV irradiation.

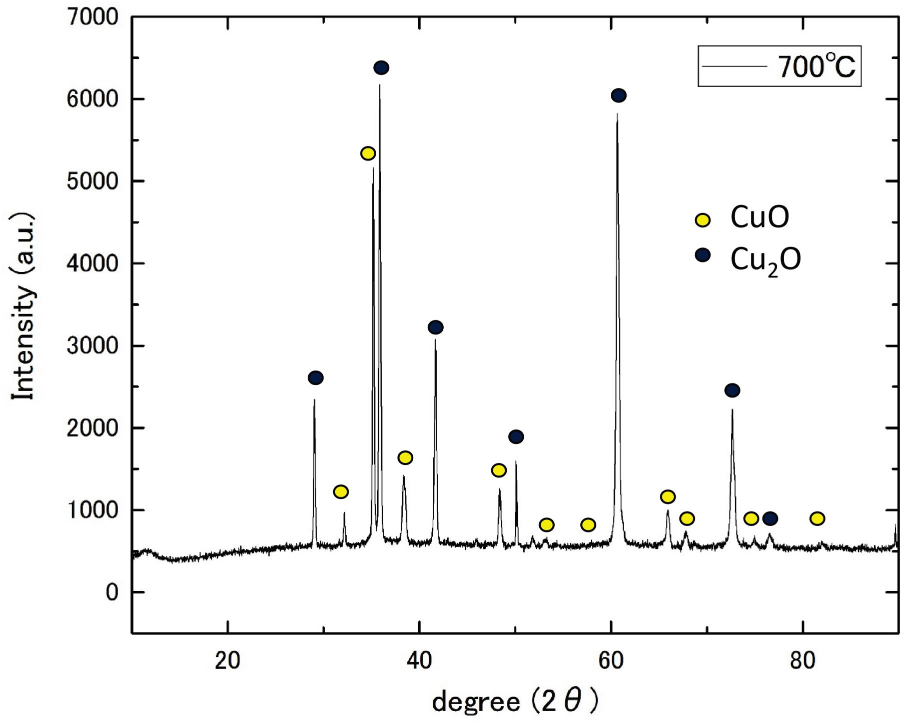

Supplementary Figure S4. XRD patterns of the sample after been annealed at 700 °C.
